# Optical Properties of Some New Azo Photoisomerizable Bismaleimide Derivatives

**DOI:** 10.3390/ijms12096176

**Published:** 2011-09-21

**Authors:** Anton Airinei, Nicusor Fifere, Mihaela Homocianu, Constantin Gaina, Viorica Gaina, Bogdan C. Simionescu

**Affiliations:** 1 “Petru Poni” Institute of Macromolecular Chemistry, 41A Aleea Grigore Ghica Voda, 700487 Iasi, Romania; E-Mails: nfifere@yahoo.com (N.F.); michalupu@yahoo.co.uk (M.H.); cgaina@icmpp.ro (C.G.); vgaina@icmpp.ro (V.G.); bcsimion@icmpp.ro (B.C.S.); 2 Department of Natural and Synthetic Polymers, “Gheorghe Asachi” Technical University, Iasi 700050, Romania

**Keywords:** electronic absorption spectra, azo chromophore, bismaleimide, photoisomerization, thermal cis-trans relaxation

## Abstract

Novel polythioetherimides bearing azobenzene moieties were synthesized from azobismaleimides and bis-2-mercaptoethylether. Kinetics of trans-cis photoisomerization and of thermal conversion of cis to trans isomeric forms were investigated in both polymer solution and poly(methyl methacrylate) doped films using electronic absorption spectroscopy. Thermal recovery kinetics is well described by a two-exponential relation both in solution and polymer matrix, while that of low molecular weight azobismaleimide fit a first-order equation. The photoinduced cis-trans isomerization by visible light of azobenzene chromophores was examined in solution and in polymer films. The rate of photoinduced recovery was very high for azobismaleimides.

## 1. Introduction

Organic materials based on the azobenzene moiety have received significant attention because of their photoresponsive properties that results from the trans-to-cis and cis-to-trans isomerizations of azo chromophores [1–7]. Reversible transformation between more stable thermodynamically trans-azobenzene and less stable cis-azobenzene can be monitored by UV or visible light irradiation or thermally in the dark. The photoisomerization process can be followed by monitoring the intensity of the absorption band of the trans isomers at around 350 nm while the cis isomers show a less intense absorption band at longer wavelengths [1,6]. In this way the light response becomes of special importance in order to control at will the molecule configuration. Azobenzene-photoresponsive polymers were widely studied because of their promising applications in a number of key fields such as optical switching and data storage, liquid crystal displays, molecular machines, surface relief gratings, nanodevices, nonlinear optics [8–16].

The photosensitive properties of azobenzene-containing polymers depend on the chemical structure of polymer chain, azo chromophore types, positions to which azo chromophores are attached. In this context, the understanding of the factors controlling the isomerization mechanisms and reaction kinetics of azo chromophore can play a prominent role in order to design novel photoreactive materials. Also, the photoisomerization of azobenzene and its derivatives influences their physical characteristics namely viscosity, wettability or aggregation behavior due to the structural changes occurring between trans and cis isomers.

The bismaleimide resins have attracted much attention in the fields of advanced composites due to their excellent processing characteristics without the formation of volatile by-products and outstanding thermomechanical and flammability behavior on the finally cured state. Although a variety of bismaleimide derivatives was synthesized for preparation of bismaleimide resins [17,18], there is a lack of information about their photochemical behavior.

In this paper the preparation of some azobismaleimides and polythioetherimides bearing azobenzene units by Michael addition reaction was performed. The photoisomerization behavior of azobenzene moieties in bismaleimides, as well as attached covalently to a polymer chain or incorporated in a polymer matrix, was explored in solution and in polymer films. Thermal cis-trans isomerization process of azobismaleimide embedded in poly(methyl methacrylate) matrix have been discussed over a range of temperatures and the isomerization kinetics was found to be determined by environment-imposed constraints.

## 2. Experimental Section

### 2.1. Materials

Maleic anhydride, bis-2-mercaptoethylether, triethylamine, acetone were purchased from Aldrich and utilized without further purification. Solvents for spectral analysis were in spectrophotometric grade (Aldrich). Poly(methyl methacrylate) was employed as received from Aldrich with *M*_w_ = 120.000 (*T*_g_ = 99 °C). 2,4-Diamino-2′-methylazobenzene (DA2MAB) and 2,4-diamino-4′-methylazobenzene (DA4MAB) were prepared by an one-step diazonium coupling reaction of o- or p-toluene diazonium chloride with m-phenylenediamine [19].

### 2.2. Azobismaleimide Synthesis

Azobismaleimides ABM 1 and ABM 2 were obtained from DA2MAB or DA4MAB (1 mol) and maleic anhydride (2 mol) in dry acetone by a two-step method [20,21]. A typical procedure is presented below. To a solution of maleic anhydride (1.96 g, 0.02 mol) in acetone (80 mL), a solution of DA2MAB (2.26 g, 0.01 mol) in acetone (50 mL) was added. The reaction mixture was stirred for 1.5 h at room temperature and then anhydrous magnesium acetate (1.2 mg), triethylamine (1.14 mL) and acetic anhydride (2.14 mL) were added. The reaction mixture was refluxed for 4 h. The excess of acetone was removed in vacuum and the solid residue (ABM 1) was washed several times with water and then recrystallized from ethanol, *m*_p_ = 135–138 °C. The yield was 70%. Elemental analysis: Calculated (%): C 65.28; H 3.65; N 14.50; Found (%): C 65.42; H 3.48; N 14.83. FT-IR (KBr, cm^−1^): 3090, 1719, 1590, 1510, 1443, 1368, 1148, 830, 767, 692. ^1^H NMR (400 MHz, CDCl_3_, δ (ppm)): 2.69 (s, 3H, CH_3_); 6.88 (d, 4H; CH = maleimide protons), 7.14–7.32 (m, 4H, aromatic), 7.65 (d, 2H, aromatic), 7.94 (s, 1H, aromatic).

ABM 2 was prepared as mustard solid crystals by recrystallization from 1,2-dichlorethane/ethanol, *m*_p_ = 255–258 °C. The yield of compound ABM 2 was 83%. Elemental analysis: Calculated (%): C 65.28; H 3.65; N 14.50; Found (%): C, 64.96; H, 3.73; N, 14.78. FT-IR (KBr, cm^−1^): 3090, 1715, 1600, 1991, 1444, 1373, 1144, 830, 690. ^1^H NMR (400 MHz, CDCl_3_, δ (ppm)): 2.46 (s, 3H, CH_3_), 6.88 (d, 4H, CH = maleimide protons), 7.26 (d, 2H, aromatic), 7.60–7.69 (d, 4H, aromatic), 7.98 (s, 1H, aromatic).

### 2.3. Polymer Synthesis

To a solution of ABM 2 (0.89 g, 2.3 mmol), in freshly distilled m-cresol (8 mL), bis-2-mercaptoethylether (0.3 mL, 2.3 mmol) and two drops of triethylamine were added in a 50 mL three-necked flask equipped with a magnetic stirrer, thermometer and condenser. The reaction mixture was stirred at 70 °C for 10 h. The polymer was obtained by pouring the reaction mixture into 50 mL methanol acidified with glacial acetic acid. The precipitate was washed for several times with methanol and then extracted overnight with methanol using a Soxhlet extractor and dried in a vacuum oven at 60 °C for 14 h.

### 2.4. Measurements

FT-IR absorption spectra were taken on a Bruker Vertex 70 spectrometer equipped with a golden gate single reflection ATR accessory. ^1^H NMR spectra were collected on a Bruker Avance DRX spectrometer using DMSO-d6 and CDCl_3_ as solvents and tetramethylsilane as the internal standard. Melting points were as determined with a Gallenkamp hot-block point apparatus.

UV-Vis absorption spectra were measured by SPECORD 200 Analytik Jena and UV-3600 Shimadzu spectrophotometers. The samples were dissolved in spectroscopic solvents and maintained in the dark for 48 h before the absorption spectra were recorded. All measurements were performed at room temperature. Photoirradiation was performed in solution or in polymer films using a 500 W high pressure mercury arc lamp, and suitable glass filters were utilized to obtain the irradiation light. Photochromic reactions were monitored following the changes in ultraviolet-visible absorption spectra. The samples were maintained at constant temperature using a temperature controller. The polymer films were prepared on quartz substrate by casting from dichloroethane solution by mixing equal amounts of 5% poly(methyl methacrylate) (PMMA) solution and 0.1% solution of azobenzene compound ABM 2. The polymer films were dried under reduced pressure at 50 °C for 24 h.

Atomic force microscopy (AFM) measurements were achieved with a SOLVER PRO-M system (NT-MDT, Russia) in semicontact mode. Commercially available Si cantilevers with a mean force constant of 11.5 N/m were utilized.

## 3. Results and Discussion

**Scheme 1:**
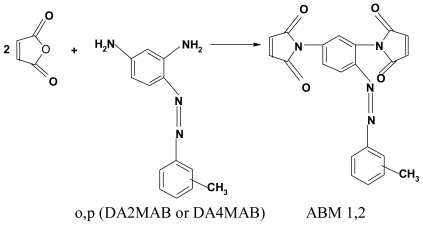
Chemical structure of the studied azobismaleimides.

Azobismaleimides containing azobenzene groups ABM 1 and ABM 2 were obtained in high yield by the condensation reaction of azoaromatic diamines (DA2MAB or DA4MAB) with maleic anhydride followed by *in situ* cyclodehydration with a mixture of acetic anhydride and triethylamine [20] (Scheme 1).

**Figure 1:**
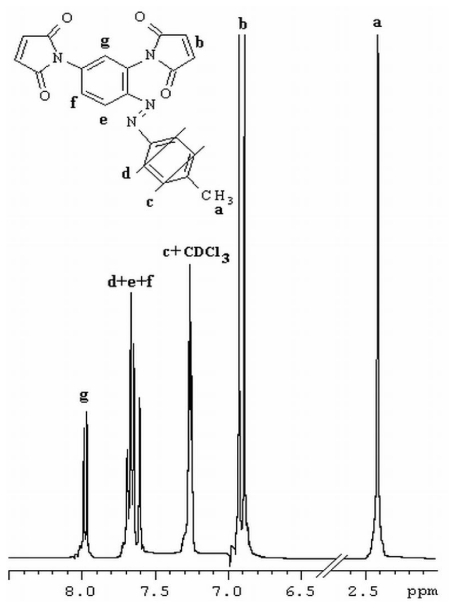
^1^H NMR spectrum of azobismaleimide ABM 2 in CDCl_3_.

**Scheme 2:**
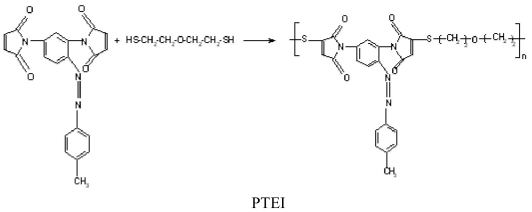
Chemical structure of the studied azopolymer.

FT-IR and ^1^H-NMR spectroscopy and elemental analysis supported the proposed structures. FT-IR spectra of azobismaleimides evidenced the presence of absorption bands at 1725, 1720, 1368–1373, 1148–1149 and 690–692 cm^−1^ attributed to imide cycle, 830 cm^−1^ corresponding to out plane hydrogen deformation of cis-disubstituted double bond of maleimide groups conjugated with carboxyl groups. ^1^H-NMR spectra of azobismaleimide ABM 2 (Figure 1) exhibited the chemical shifts characteristic for CH_3_ signal at 2.46 ppm, maleimide protons at 6.88 ppm, aromatic protons appearing as doublet at 7.26 ppm and as singlet at 7.98 ppm. Chain extension of bismaleimides with bisthiols can be used in order to obtain polyimidisulfides [22,23]. The reaction of an equimolecular mixture of bismaleimide ABM 2 and bis-2-mercaptoethylether gives polythioetherimide (PTEI) containing azobenzene groups (Scheme 2).

FT-IR absorption spectrum of polymer displayed absorption bands at about 1715–1720, 1364–1373, 1175 and 707 cm^−1^ due to imide group, at 2938, 2856 and 1435 cm^−1^ assigned to aliphatic moiety, at 1206 cm^−1^ due to C-O-C group of bisthiols and at 1100 cm^−1^ corresponding to C-S-C group.

Azobismaleimides ABM 1 and ABM 2, and polymer PTEI belong to the azobenzene type molecules, according to Rau classification [1,24]. Their electronic absorption spectra exhibit two characteristic absorption bands related to the intense π → π* transition of trans-form azobenzene at about 345.5 nm (ε = 21740 L mol^−1^ cm^−1^ (*N*,*N*-dimethylformamide (DMF)) and low absorption at 442 nm (ε = 1270 L mol^−1^ cm^−1^) which originates from an *n* → π* transition for ABM 2. Azobismaleimide ABM 1 exhibits an absorption band at 342 nm with ε = 18160 L mol^−1^ cm^−1^.

**Figure 2:**
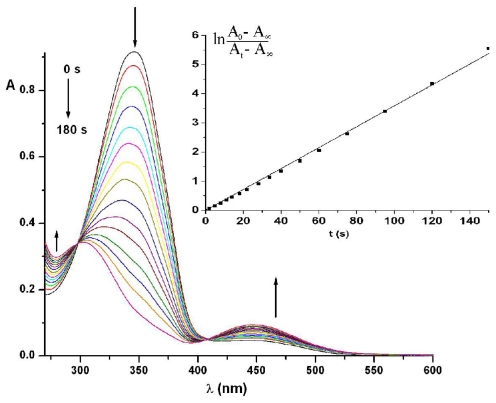
Dependence of UV-Vis absorption spectra of ABM 2 in *N*,*N*-dimethylformamide (DMF) solution on UV irradiation. Inset shows a first-order plot for trans-cis photoisomerization. Arrows indicate the directional changes of the spectra.

Under 365 nm light irradiation the azobismaleimides ABM 1 and ABM 2 undergo isomerization from trans to cis forms of azobenzene chromophore. As it can be seen from Figure 2, the intensity of absorption band at about 345.5 nm (π → π* transition) of azobismaleimide ABM 2 decreased progressively, while the absorption band corresponding to the n → π* transition in cis isomer around 442 nm increased with irradiation time, suggesting that the isomerization of azobenzene chromophore from trans to cis form until a photostationary state was reached. The degree of photoisomerization at the photostationary state, R, was evaluated from the relation: R = (*A*_0_ − *A*_∞_)/*A*_0_ × 100, where A_0_ is the initial absorbance and A_∞_ is the absorbance at the photostationary state. It should be noted that the photoisomerization process lasts for about 200 s with a conversion in cis isomer of 0.85. The sharp isosbestic points at 297 and 410 nm showed that the azobenzene isomerization reaction is a single step process where only two species (trans and cis) are involved.

Photoisomerization kinetic data were fitted to equation:

(1) $$\text{ln}\;[(A_{0}-A_{\infty })/(A_{\text{t}}-A_{\infty })]=kt$$

where *A*_0_, *A*_t_ and *A*_∞_ represent absorbance before irradiation, at irradiation time *t* and at the photostationary state [25]. The rate constant, *k*, is given by the following relation: *k* = *I*_0_(ɛ_t_φ_tc_ + ɛ_c_φ_ct_)ln10 + *k*_ct_, where φ_tc_ and φ_ct_ denote the quantum yields of the trans-cis and cis-trans photoisomerization reactions, ɛ_t_ and ɛ_c_ denote the molar absorptivities of the trans and cis isomers at the irradiation wavelength, I_0_ is the incident radiation intensity and k_ct_ is the rate constant for thermal isomerization. Its value is very small at room temperature and this term was neglected. Plot of ln[(*A*_0_ − *A*_∞_)/(*A*_t_ − *A*_∞_)] *versus* irradiation time for ABM 2 gives the value of *k* from its slope (Figure 2, inset). A value of (3.31 ± 0.03) × 10^−2^ s^−1^ was obtained for the photoisomerization rate constant of ABM 2 in DMF solution.

**Figure 3:**
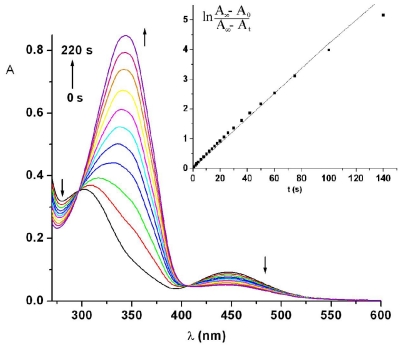
Time evolution of electronic absorption spectra of ABM 2 in DMF under 436 nm light irradiation. Inset shows cis-trans isomerization kinetics. Arrows indicate the directional changes of the spectra.

After saturated UV light irradiation, the cis-trans isomerization could be induced by irradiation with visible light (436 nm) or thermally. The bismaleimide ABM 2 in DMF was exposed under visible light of 436 nm and the changes in the electronic absorption spectra were depicted in Figure 3.

The reversion from cis-isomer to trans-isomer leads to a gradual increase in the intensity of the absorbance at 345.5 nm. The back cis-trans isomerization takes about 200 s to achieve the equilibrium, but the absorption spectrum was completely restored to the starting one after 24 h. Fitting of the experimental data to a first-order kinetics (Figure 3, inset) is valid for the first stages of the reaction after irradiation times higher than 90 min a deviation from the straight line occurred. The estimated value of the rate constant for the cis-trans isomerization corresponding to the linear part under blue light was (4.38 ± 0.03) × 10^−2^ s^−1^ being of the same order of magnitude as photoisomerization process.

Similar spectral changes were observed for azobismaleimide ABM 1 in DMF solution when it was exposed to 365 nm light irradiation. In this case the photostationary equilibrium was attained after 300 s at a photoconversion rate of only 60%, indicating a dependence of photostationary state composition on the chemical structure of the maleimide. The rate constant of photoisomerization process for ABM 1 was about two times lower than that of ABM 2.

**Figure 4:**
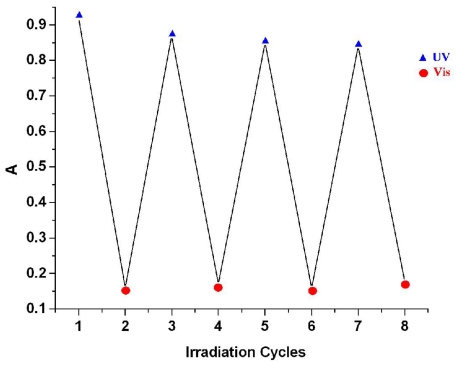
UV-Vis switch cycles of ABM 2 in DMF solution upon alternate irradiation with the UV/blue light.

Preliminary tests show that the intensity of the absorption band at 345.5 nm (ABM 2) can be reversibly switched by alternate irradiation with UV and visible light (436 nm) during several cycles of UV light induced trans-cis and blue light induced cis-trans-isomerization (Figure 4).

The absorbance values and the positions of the absorption band were maintained without noticeable change. Compared to ABM 1, azobismaleimide ABM 2 has higher isomerization efficiency, because its cis content in photostationary state was about 85%, followed by the rapid recovery induced by blue light. The high proportion of cis isomer after irradiation with UV light and rapid recovery with visible light can be important factors for application aspects such as photoswitching.

**Figure 5:**
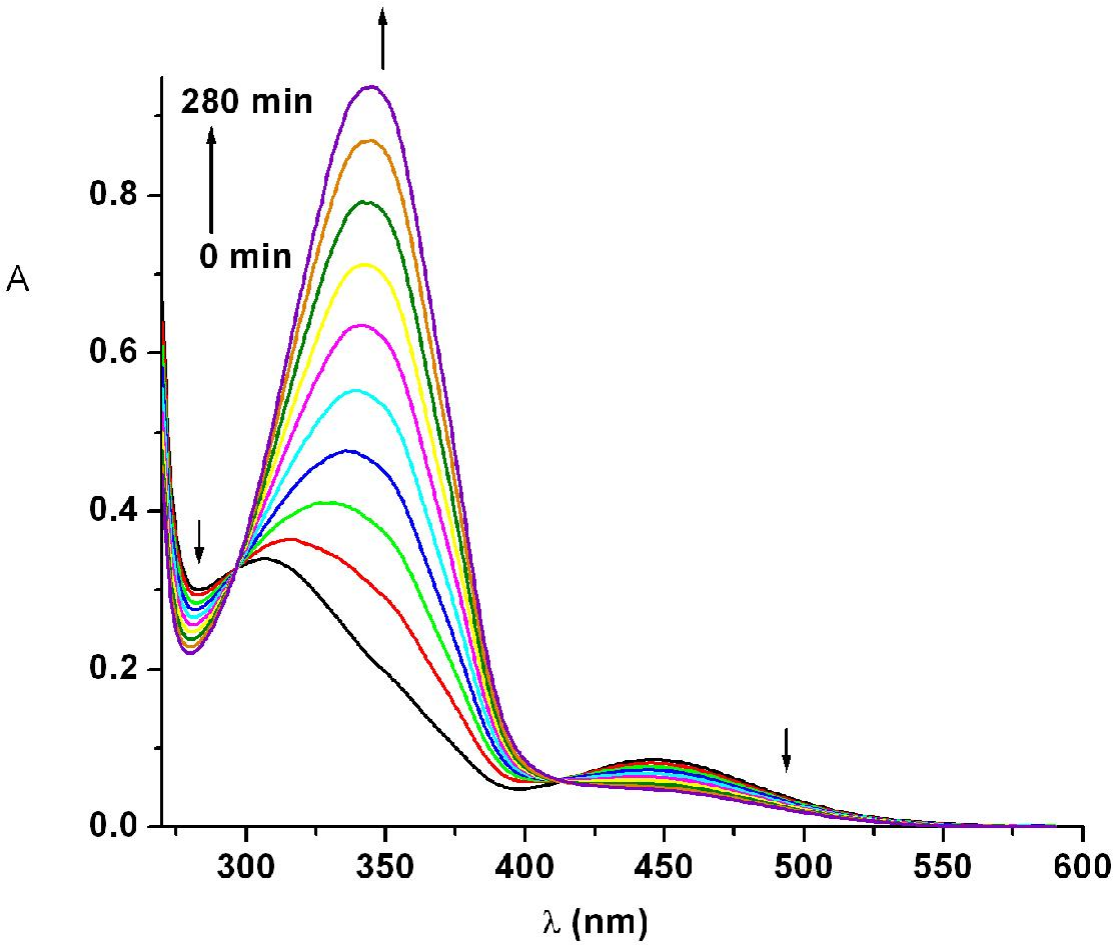
Absorption spectral changes of ABM 2 in DMF during thermal recovery at 60 °C.

**Figure 6:**
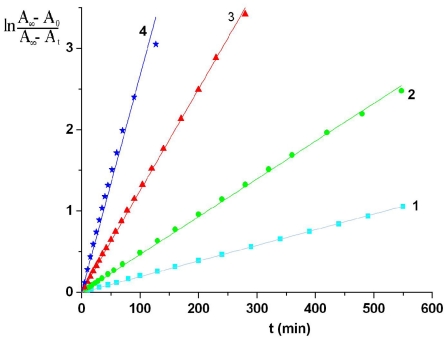
Kinetics of thermal cis-trans isomerization of ABM 2 in DMF solution: (1) 40 °C; (2) 50 °C; (3) 60 °C; (4) 70 °C.

Thermal cis-trans isomerization was followed by monitoring the intensity of absorption at 345.5 nm of ABM 2 in DMF solution primarily exposed to 365 nm light to obtain photostationary state. During the dark keeping at a certain temperature the absorbance at 345.5 nm increases remarkably while the absorbance at about 440 nm decreases, indicating that azobenzene moieties revert thermally from cis to trans forms (Figure 5). The kinetics of cis-trans reverse thermal isomerization of ABM 1 and ABM 2 in DMF solutions were fitted satisfactorily to the Equation (2):

(2) $$\text{ln}(A_{\infty }-A_{0})/(A_{\infty }-A_{\text{t}})=k_{\text{ct}}\;t$$

where *A*_0_, *A*_t_ and *A*_∞_ are the absorbances at 345.5 nm at times 0, *t* and infinite, respectively and *k*_ct_ is the rate constant. Typical first-order plots according Equation (2) for azobismaleimide ABM 2 at different temperatures are shown in Figure 6.

**Figure 7:**
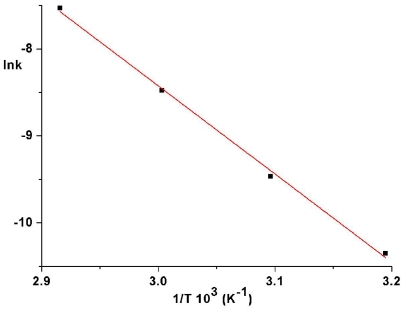
Arrhenius plot for thermal cis-trans isomerization rate constants for ABM 2.

The values of *k*_ct_ are evaluated from the slopes of plots ln[(*A*_∞_ − *A*_0_)/(*A*_∞_ − *A*_t_)] as a function of time. An Arrhenius plot of the thermal isomerization rate constants of ABM 2 gives a straight line (Figure 7) and allowed us to estimate an activation energy value of (19.48 ± 0.66) kcal/mol and a preexponential factor *Z* = 1.25 × 10^9^ s^−1^. This value of activation energy is in good agreement with typical values for other azobenzene derivatives [1,6,26,27].

**Figure 8:**
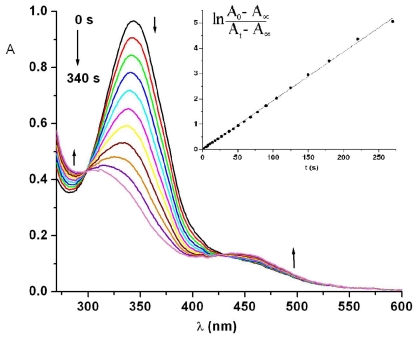
Photoisomerization of azopolythioetherimide (PTEI) in DMF solution upon irradiation at 365 nm. Inset indicates the trans-cis photoisomerization kinetics.

Photoisomerization of azopolymer PTEI was carried out in the DMF solution by UV light irradiation. Figure 8 reveals the changes in electronic absorption spectra of polymer PTEI in DMF solution during UV irradiation.

The UV-Vis absorption spectra are characterized by a strong π → π* transition of the trans azobenzene chromophore located at 343 nm and a weak absorption band at 445 nm assigned to a *n* → π* transition. A photostationary state was obtained after 340 s. Analysis of spectral changes leads to the conclusion that the DMF solution of PTEI in the photostationary state after UV light irradiation contains about 65% of azobenzene moieties in cis form and approximately 35% in trans form. It was noticed that azopolymer required more time to reach the photostationary state and a less amount of cis-isomer fraction in photostationary state was obtained indicating an inhibition of the photoisomerization due to a relatively small free volume determined by the polymer chains. Photoisomerization of the azopolymer in solution exhibits isosbestic points, at 298 and 430 nm, respectively, suggesting uniform photoreactions.

The kinetics of photoisomerization of PTEI in DMF were plotted in Figure 8, inset and obey to first-order kinetics with a smaller rate constant about (2.14 ± 0.01) × 10^−2^ s^−1^ as compared to azobismaleimide ABM 2. The same spectral pattern was maintained when the back reaction was performed by irradiation with visible light (436 nm) just like in the case of ABM 2. The first-order kinetics of recovery by blue light irradiation deviated from the straight line for longer irradiation times. The rate constant estimated from the first-order part of the plot was calculated to be (4.71 ± 0.07) × 10^−2^ s^−1^.

**Figure 9:**
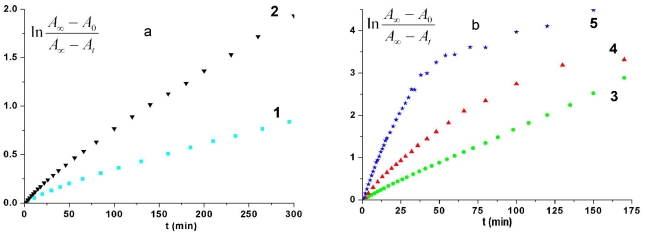
Kinetic data for cis-trans thermal recovery of azo polymer PTEI in DMF solution: (1) 40 °C; (2) 50 °C; (3) 60 °C; (4) 70 °C; (5) 80 °C.

**Figure 10:**
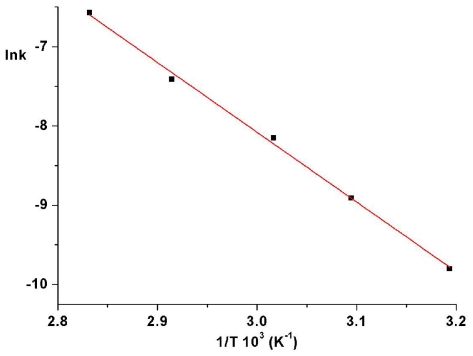
Arrhenius plot for thermal recovery rate constant of azopolymer PTEI in solution.

The thermal cis-trans recovery of the irradiated sample PTEI in DMF was investigated at different temperatures. The intensity of absorption band around 342 nm was slowly restored to the trans initial state of PTEI at about 300 min when the recovery was carried out at 60 °C. It is known the thermal cis-trans recovery of azobenzene moieties usually follows a first-order kinetics in solution. However, kinetics curves corresponding to thermal cis-trans isomerization of PTEI in DMF solution deviated from a straight line (Figure 9) as for low-molecular compound AMB 2. The thermal isomerization proceeded faster in the beginning of isomerization reaction and then was followed by a slow process. Thus, kinetic curves corresponding to thermal cis-trans isomerization of PTEI in DMF solution did not follow a simple first-order pathway (Figure 9). The experimental data can be analyzed by a first-order kinetics with two components described by the following equation:

(3) $$\left(A_{\infty }-A_{\text{t}})/(A_{\infty }-A_{0})=\alpha \;\text{exp}\;(-k_{\text{f}}\;t)+(1-\alpha )\;\text{exp}(k_{\text{s}}t\right)$$

where *k*_f_ and *k*_s_ are the rate constant of the fast and slow relaxation processes and α represents the preexponential term of the fast process. The value of α was 0.18 when the thermal recovery was conducted at 50 °C. Fitting the experimental data to relation (3) a separate calculation of Arrhenius parameters for fast and low processes was carried out. As shown in Figure 9, all the plots deviate from a straight line suggesting that the reaction cannot be described by a single-exponential recovery process by comparison with low-molecular compound ABM 2. Figure 9 reveals that fraction of fast component of thermal isomerization is smaller for lower temperatures (α = 0.16 at 40 °C) and it rapidly increases with increasing temperature (α = 0.66 at 60 °C) (Figure 9b), consistent with higher mobilities of the polymer chains which lower the resistance to structure change in the isomerization. The Arrhenius plot of the rate constants for azo polymer PTEI is given in Figure 10. From the slope of Arrhenius plot of initial isomerization rate the apparent activation energy (*E*_a_) and preexponential factor (*Z*) were estimated: *E*_a_ = (17.83 ± 0.48) kcal/mol and *Z* = 1.41 × 10^8^ s^−1^, respectively. In the case of azopolymer PTEI the activation energy is lower relative to the azobismaleimide ABM 2 due to the presence of constrained cis-isomers in the polymer chain leading to a decrease of activation energy for cis-trans izomerization process because of the instability of cis-isomers [28].

**Figure 11:**
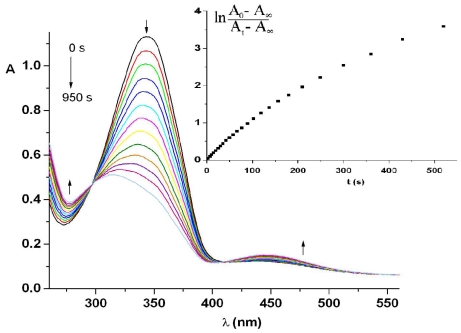
Spectral changes of poly(methyl methacrylate) (PMMA) film containing azochromophore under UV irradiation (365 nm). Inset shows the trans-cis photoisomerization kinetics.

The photochromic behavior of azobismaleimide ABM 2 induced by photoirradiation at room temperature was investigated in PMMA amorphous films. The absorption spectrum of ABM 2 in PMMA matrix showed two bands centered around 343 and 444 nm, respectively, attributed to the π → π* and *n* → π* electronic transition of azo chromophore (Figure 11). When arrived at the photostationary state (after about 750 s) a photoisomerization degree of 65% was obtained similarly to the azopolymer PTEI in solution.

In the evolution of photoprocess, isosbestic points at 296 and 410 nm were observed. In this case the time profile of the absorbance variation of trans isomer during UV irradiation exhibited a deviation from a first-order kinetics (Figure11, inset) in contrast with the photoisomerization in solution which obey a simple first-order kinetics. Photoisomerization of azobenzene moieties in polymer film can be analyzed by a first-order kinetics with two components [29,30], according to Equation (4):

(4) $$\left(A_{0}-A_{\infty })/(A_{\text{t}}-A_{\infty })=\alpha \;\text{exp}\;(-k_{\text{f}}\;t)+(1-\alpha )\;\text{exp}\;(-k_{\text{s}}\;t\right)$$

where *k*_f_ and *k*_s_ are the rate constants for the fast and slow components of the photoisomerization process, α is the fraction of faster photoisomerization to total conversion. A value of 0.19 was found for parameter α. The combination of fast and slow processes in the photoisomerization of azobenzene chromophores in polymer film is determined by the distribution of free volume size in the polymer matrix. The free volume size in the proximity of the azobenzene chromophore corresponding to the fast and slow processes is ascribed to larger and smaller volumes than the critical size [31]. The volume required for isomerization of azobenzene unit was evaluated to be 127 Å^3^ [32].

The rate of trans-cis photoisomerization for azobenzene chromophore embedding in polymer matrix was evidently lower than that for polymer PTEI and azobismaleimide ABM 2 in solution. The trans-cis isomerization reaction occurred in thin film through isosbestic points at 295 and 415 nm. The rate constant of ABM 2 in polymer film was estimated to be (1.15 ± 0.02) × 10^−2^ s^−1^ in the fast photoisomerization process and (5.66 ± 0.03) × 10^−3^ s^−1^ for the slow process. The lower rate constant in PMMA films for the fast isomerization process can be determined by the polarity of PMMA matrix. The influence of the polarity was confirmed by the hypsochromic shift of the absorption maximum of azo moiety to 343 nm in polymer film relating to the azobismaleimide ABM 2. Also, it can be envisaged that more crowded environment for polymer slows down the rate of the photoisomerization process in comparison with DMF solution.

The polymer film was irradiated with 436 nm visible light in order to determine the backward cis-trans isomerization. The same absorption spectral pattern was obtained for the thin polymer film as that of polymer PTEI in DMF solution. The kinetics of the cis-trans isomerization also deviated from a first-order kinetics. However, the time taken to obtain photostationary state was higher as compared to solution phase.

**Figure 12:**
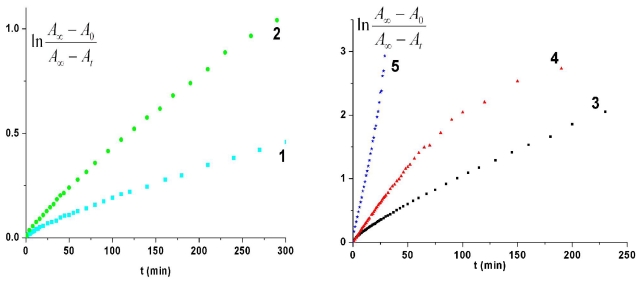
Thermal cis-trans isomerization of ABM 2 in PMMA film for different temperatures: (1) 40 °C; (2) 50 °C; (3) 60 °C; (4) 70 °C; (5) 80 °C.

Kinetic curves corresponding to thermal cis-trans recovery of ABM 2 in PMMA films cannot be fitted with a single exponential function (Figure 12). Applying a biexponential kinetics (3) the thermal relaxation of the cis-isomer in film can be described using our experimental data and a value of 0.18 was obtained for α. Deviations from a first-order kinetics are generally determined by the inhomogeneous distribution of local free volume in polymer matrix [33,34]. The bimodal kinetics observed for thermal cis-trans isomerization of azobenzenes chromophores in polymer films can be interpreted as being due to the trapping of some of cis-isomers in a strained conformation which isomerize more rapidly to the trans state than the relaxed cis remaining isomers [33,35]. The two rate constants for ABM 2 in PMMA films were (7.68 ± 0.14) × 10^−5^ and (5.00 ± 0.06) × 10^−5^ s^−1^, respectively at 50 °C. The values of cis-trans rate constants were not different from other systems containing azobenzene moieties incorporated in PMMA. The rate constants determined for thermal relaxation of 4-dimethylaminoazobenzene in PMMA matrix at 30 °C were found out to be 1.83 × 10^−4^ and 8.67 × 10^−5^ s^−1^, respectively [36]. In the case of thermal isomerization of 4-(N-maleimido) azobenzene chromophore in polystyrene films at 50 °C the two rate constants corresponded to the following values: 1.34 × 10^−4^ and 6.43 × 10^−5^ s^−1^ [37]. The fast isomering part of total cis-isomer concentration increased with the increase of temperature (Figure 12) due to the different relaxation mechanisms of the two cis-species which depends directly on the local segmental mobility around chromophore. The values of activation energy and preexponential factor corresponding to the first process of cis-trans recovery estimated from Arrhenius plot were (20.82 ± 0.82) kcal/mol and 8.43 × 10^9^ s^−1^, respectively. In this case the activation energy for polymer film is higher compared to solution and the catalytic effect of polymer on thermal cis-trans isomerization was not observed. Similar value of Ea was obtained for other azobenzene chromophores in PMMA matrix [38,39].

**Figure 13:**
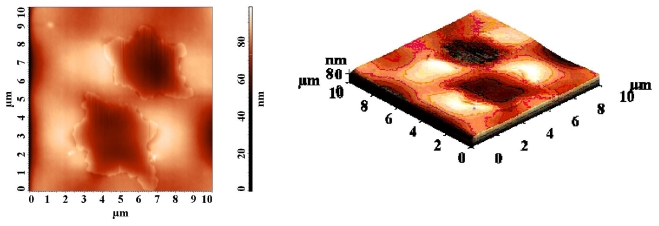
Atomic force microscopy images of PMMA film containing azobismaleimide ABM 2.

**Figure 14:**
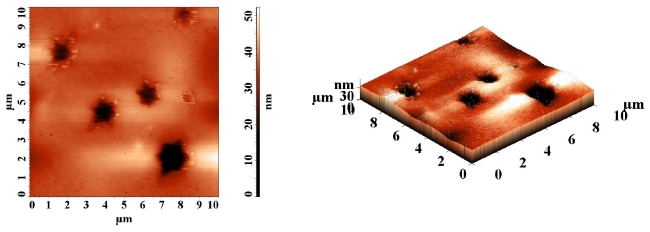
Topographical AFM image of a PMMA/ABM 2 films after UV irradiation.

The topographic characteristics of PMMA containing 0.2% azobismaleimide ABM 2 were discussed using atomic force microscopy (AFM) in tapping mode. Figures 13 and 14 present the two- and three- dimensional morphology of the polymer film bearing azo chromophores before and after UV irradiation when the photostationary state was attained.

Before UV irradiation a number of partially elongated hexagonal microdomains were observed (Figure 13) with average width of 4 μm and depth of *ca*. 10–15 nm. The root mean square roughness σ_RMS_ = 8.4 nm in this image. The images were recorded in different points of the film surface in order to check the reproducibility. Generally, the same morphology pattern was maintained after UV irradiation (Figure 14) but the average depth of hexagonal structure was increased to *ca*. 20 nm and its width was 1.7 μm. After UV irradiation the mean square roughness becomes 4.83 nm. The observed reduction of the surface roughness (Figure 14) could be due to the fluidification effect that the cis isomers have on the polymer. This effect is very probably linked with the more isotropic shape of the cis conformer of the azobenzene unit [40] providing for a better surface smoothing.

## 4. Conclusions

Novel polythioetherimide bearing azobenzene moieties were synthesized by the reaction between azobismaleimides and bis-2-mercaptoethylether. The trans-cis photoisomerization reaction and cis-trans recovery of azobismaleimides, azopolythioetherimide in DMF solution and azobismaleimide in PMMA matrix were investigated by irradiation with UV light and visible light (436 nm), respectively. Azobismaleimides have shown a faster trans-cis photoisomerization ability in relation to azopolymer. Thermal cis-trans isomerization of azobenzene chromophores was investigated in DMF solution as well as in PMMA film. The thermal recovery of cis isomers in polymer film has been fitted by a sum of two first-order processes, a fast and a slow one. This paper reports for the first time the spectrokinetic studies of photochemical isomerization of azobenzene chromophore in polythioetherimide.
